# Atypical evening cortisol profile induces visual recognition memory deficit in healthy human subjects

**DOI:** 10.1186/1756-6606-1-4

**Published:** 2008-08-21

**Authors:** Heather Gilpin, Daniel Whitcomb, Kwangwook Cho

**Affiliations:** 1 Biomedical Science, University of Sheffield, Sheffield, S10 2TN, UK; 2 Henry Wellcome Laboratories for Integrative Neuroscience and Endocrinology, UK; 3 The MRC Centre for Synaptic Plasticity, University of Bristol, Bristol, BS1 3NY, UK

## Abstract

**Background:**

Diurnal rhythm-mediated endogenous cortisol levels in humans are characterised by a peak in secretion after awakening that declines throughout the day to an evening trough. However, a significant proportion of the population exhibits an atypical cycle of diurnal cortisol due to shift work, jet-lag, aging, and mental illness.

**Results:**

The present study has demonstrated a correlation between elevation of cortisol in the evening and deterioration of visual object recognition memory. However, high evening cortisol levels have no effect on spatial memory.

**Conclusion:**

This study suggests that atypical evening salivary cortisol levels have an important role in the early deterioration of recognition memory. The loss of recognition memory, which is vital for everyday life, is a major symptom of the amnesic syndrome and early stages of Alzheimer's disease. Therefore, this study will promote a potential physiologic marker of early deterioration of recognition memory and a possible diagnostic strategy for Alzheimer's disease.

## Background

Cortisol levels are maximal in the early morning and minimal in the late evening in humans [1-3]. Dysregulation in the diurnal cortisol rhythm has been associated with depression and pathological aging [4,5]. For example, age-dependent increases in evening cortisol levels have been reported in healthy subjects [6-8]. The study [6] also found a phase advance in the morning acrophase in the aged subject group. In addition, nocturnal increases in cortisol in aged subjects were correlated with significant reductions in hippocampal and temporal lobe volume [9]. Recently, a significant increase of serum cortisol levels during the evening- and night-time was found in demented patients, particularly those with Alzheimer's disease [5].

Previous studies have demonstrated that circadian-mediated cortisol levels also have an important role in cognition in an age-independent manner [10-12]. For example, frequent time-zone travellers who experience disruption to their circadian rhythm had significantly higher cortisol levels during their average working day, which was associated with cognitive deficits and right temporal lobe atrophy [10,11]. However, little is known whether an atypical rhythm of diurnal cortisol levels is associated with cognitive deficits and neurological insults. The present study analyzed the relationship between diurnal cortisol levels and cognition in healthy female subjects.

## Results

**Figure 1:**
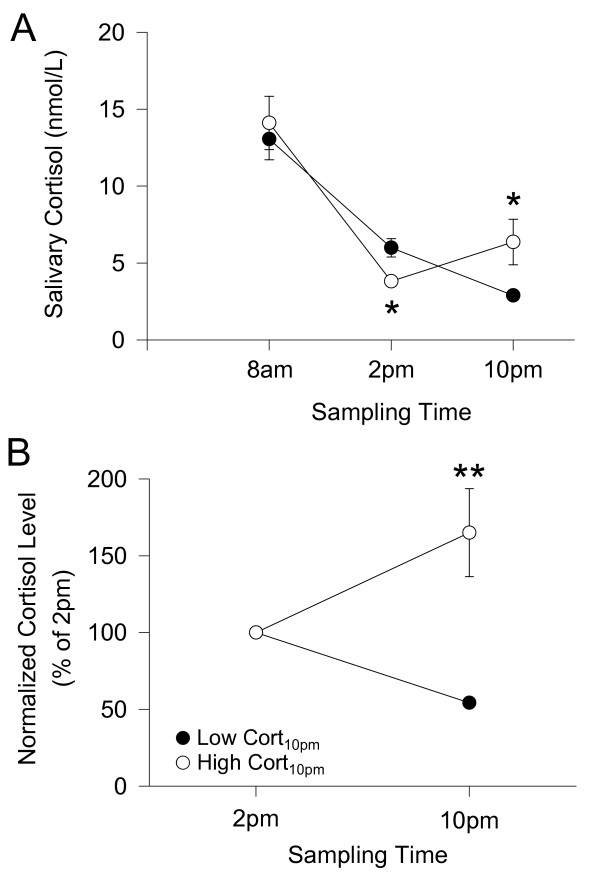
**Salivary cortisol level was analysed by means of a cortisol salivary immunoassay kit (for more details, see method)**. (A) Salivary cortisol samples were collected at three particular time points (8 am, 2 pm and 10 pm). Most subjects showed lower cortisol levels at 10 pm compared to that of 2 pm (low cortisol_10 pm_, n = 33 subjects). Eleven subjects showed higher cortisol levels at 10 pm compared to that of 2 pm (high cortisol_10 pm_). (B) Cortisol_10 pm _indicates normalized 10 pm cortisol level (percent of 2 pm cortisol level). Error bars indicate s.e.m. Significant difference at *P < 0.01, **P < 0.0001.

Subjects were 22- to 66-year-old women (Mean age = 40 yrs, age deviation = 12 yrs, n = 44 subjects) who had no neurological or psychiatric illness. Firstly, the present study analysed salivary-cortisol levels at three different time-points during the day (8 am, 2 pm and 10 pm), collected from two independent weeks (for more details see method). Since cortisol levels peak within an hour after wakeup, and decrease toward to bottom level at late evening [10], three different time saliva collecting points (8 am, 2 pm and 10 pm) will represent a major diurnal cortisol rhythm during the day. To avoid sleep/wake cycle dependent variance of diurnal cortisol rhythm, subjects were set their wakeup time at 7:30 am and sleep time at 11:00 pm. In thirty-three subjects, cortisol level at 10 pm is the lowest profile within three time points (2.9 ± 0.2 nmol/L, filled symbol, Figure 1A). In eleven subjects, however, cortisol level at 10 pm is equal to higher than that of 2 pm (6.4 ± 1.5 nmol/L, opened symbol, Figure 1A). To clarify the pattern of cortisol levels at 10 pm, data were renormalized by cortisol levels at 2 pm (low cortisol_10 pm_; 54 ± 3% of 2 pm cortisol level, n = 33, filled symbol; high cortisol_10 pm_; 165 ± 27% of 2 pm cortisol level, n = 11, opened symbol, Figure 1B). Thus, we divided subjects into two groups based on normalized 10 pm cortisol level (i.e., low cortisol_10 pm _group and high cortisol_10 pm _group). There is no difference in the mean age of the high cortisol_10 pm _group and the low cortisol_10 pm _group (low cortisol_10 pm_: 40 ± 2 years; high cortisol_10 pm_: 40 ± 4 years; P > 0.05).

**Figure 2:**
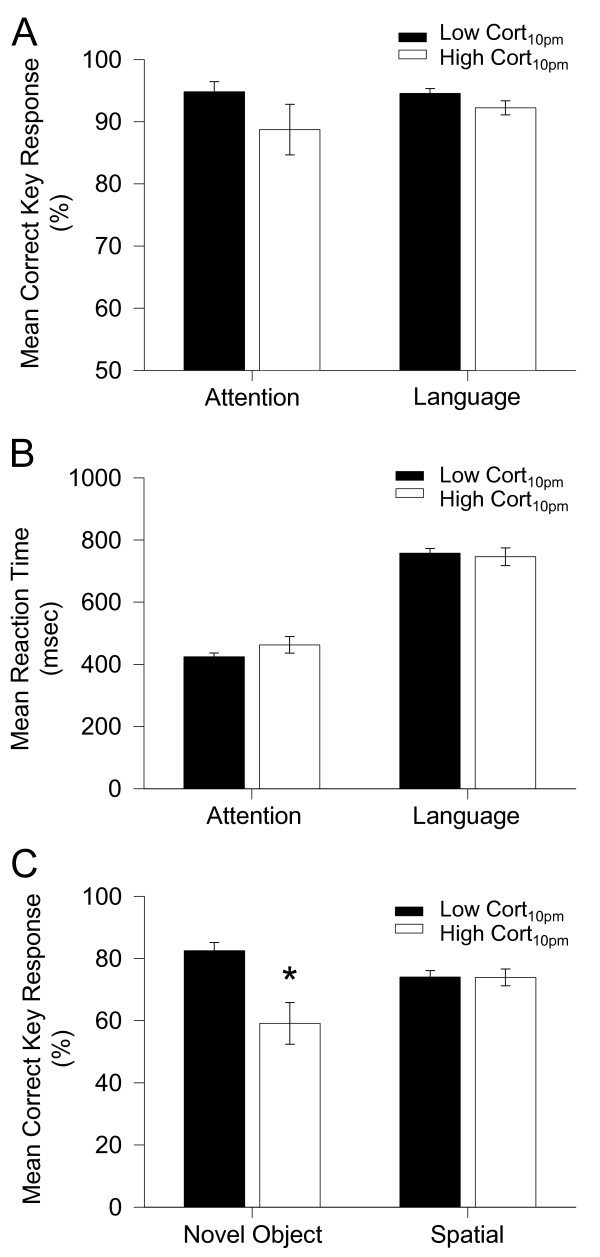
**Both the low cortisol_10 pm _subject group and the high cortisol_10 pm _subject group were compared to assess the correct key response and reaction time in cognitive performance**. (A, B) Mean (± s.e.m.) correct response and reaction time in two groups. There is no significant difference between the low cortisol_10 pm _group and the high cortisol_10 pm _group in performance in either the attention or the language task. (C) Low cortisol_10 pm _subjects show a significantly more accurate correct key response than that of high cortisol_10 pm _subjects in the novel object discrimination task. *Significant group difference at p < 0.005.

Next we analyzed whether there was any interaction between cortisol_10 pm _and cognition. In the first series of experiments, we tested attention and language in both the low cortisol_10 pm _group and the high cortisol_10 pm _group. There was no significant difference in correct key response for both the attention, (low cortisol_10 pm_: 95 ± 2% correct response, n = 32; high cortisol_10 pm_: 89 ± 4% correct response, n = 11; P > 0.05, Figure 2A) and the language task (low cortisol_10 pm_: 95 ± 1%, n = 31; high cortisol_10 pm_: 92 ± 1%, n = 9; P > 0.05, Figure 2A). Similarly, there was no significant difference in correct key response reaction time between these two groups for both the attention (low cortisol_10 pm_: 442 ± 22 msec, n = 32; high cortisol_10 pm_: 463 ± 27 msec, n = 11; P > 0.05, Figure 2B) and the language task (low cortisol_10 pm_: 758 ± 15 msec, n = 31; high cortisol_10 pm_: 746 ± 29 msec, n = 9; P > 0.05, Figure 2B).

In the next series of experiments, we analyzed visual object recognition memory and spatial memory in both subject groups (Figure 2C). In the novel object discrimination task, there was a significant difference in correct key response (low cortisol_10 pm_: 83 ± 3% correct response, n = 33; high cortisol_10 pm_: 59 ± 7% correct response, n = 11; P < 0.005, Figure. 2C). In contrast, there was no significant difference in spatial memory performance between low cortisol_10 pm _and high cortisol_10 pm _group (Figure 2C).

**Figure 3:**
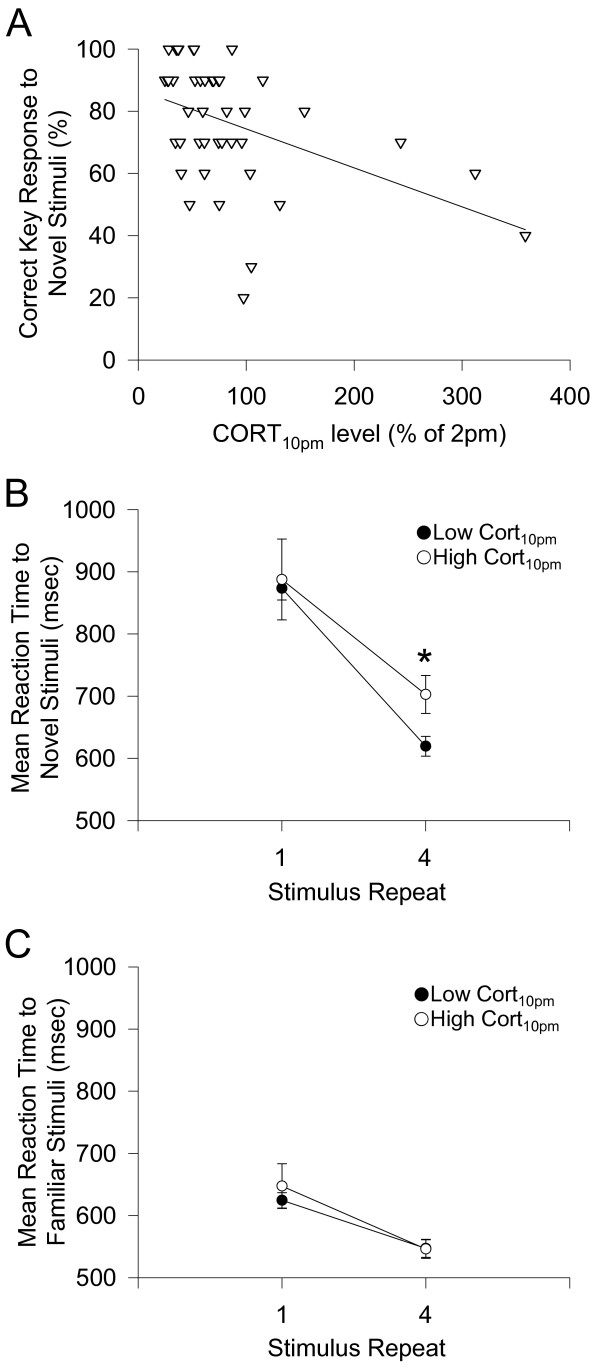
**Correlation with evening corticosterone level and familiarity discrimination**. (A) Cortisol_10 pm _level was negatively correlated with performance in the novel object discrimination across the whole cohort (r = -0.44, r^2 ^= 0.19, P < 0.01, n = 44). (B) Mean (± s.e.m.) reaction time to novel stimuli. The high cortisol_10 pm _group were significantly slower at responding to the fourth repeated presentation of a novel object. *Significant group difference at p<0.05. (C) Mean (± s.e.m.) reaction time to familiar object stimuli. There was no significant group difference (P > 0.05). Filled bars indicate low cortisol_10 pm _group, and open bars indicate high cortisol_10 pm _group.

Next the relationship between normalized cortisol_10 pm _level and percentage correct key response during the novel object discrimination task was examined by regression analysis. A significant correlation was found between these two variables (r = -0.44, r^2 ^= 0.19, P < 0.01, n = 44, Figure 3A). This initial result implies that although high evening cortisol does not influence the recognition of previously-encoded, highly recognisable objects (low cortisol_10 pm_: 97 ± 1% correct response, n = 33; high cortisol_10 pm_: 93 ± 3% correct response, n = 11; P > 0.05), it may specifically affect the processing and the ultimate familiarisation of new objects. To examine this further, we analysed the familiarity acquisition time of novel objects in both subject groups (Figure 3B). Firstly, there was no significant group difference in reaction time to the first appearance of a novel object (low cortisol_10 pm_: 873 ± 19 ms, n = 29; high cortisol_10 pm_: 888 ± 65 ms, n = 9, P > 0.05, Figure 3B). However, a significant difference was found in the reaction time to the fourth appearance of the same novel object (low cortisol_10 pm_: 619 ± 16 ms, n = 29; high cortisol_10 pm_: 703 ± 31 ms, p < 0.05, Figure 3B). This difference in reaction time was not observed at either the first (low cortisol_10 pm_: 624 ± 12 ms, n = 29; high cortisol_10 pm_: 657 ± 36 ms, n = 10, P > 0.05, Figure 3C) or the fourth presentation of a familiar object (low cortisol_10 pm_: 547 ± 14 ms, n = 29; high cortisol_10 pm_: 546 ± 15 ms, n = 10, P > 0.05, Figure. 3C).

## Discussion

The present study demonstrates a significant correlation between normalized cortisol_10 pm _level and novel object discrimination and recognition. However, there was no significant relationship between cortisol_10 pm _level and either attention or language. These results suggest no difference in either generic learning or visual perception between the two subject groups. Since the results are consistent with previous study [11], salivary cortisol level associated with the circadian rhythm may have a specific role in non-semantic cognition. As yet, we do not know whether evening cortisol level has a particularly important role in non-semantic cognition. A recent study suggests that acute stress regulates memory in a circadian rhythm-mediated manner [12]. Similarly, the current investigation also indicates that the timing of stress may be important in determining its effects on certain aspects of memory. Thus, the differentiation of circadian-mediated cortisol levels may have a critical role in the regulation of non-semantic cognition. It is still a matter of further investigation whether high cortisol_10 pm _levels are due to individual differences in diurnal rhythm or basal stress levels [13], or an indication of natural aging in normal women [6,14,15].

The diurnal pattern of cortisol is considered relatively robust but is shown to be disrupted in shift workers [16-18], transmeridian flyers [11,19] and disease states such as Alzheimer's disease [5,20] and depression [21,22]. In our study, we did notice that high cortisol_10 pm _group had lower cortisol at 2 pm but higher at 10 pm. This suggests that high cortisol_10 pm _group has a different diurnal frequency of cortisol than that of low cortisol_10 pm _group. Alternatively cortisol cycle has shifted. Interestingly, variations in the diurnal pattern of salivary cortisol have been repeatedly identified among healthy populations. In these studies, most adults have a 'normal' cortisol cycle but a subset display an 'atypical' cortisol cycle [23-25]. Therefore, some people do not have the expected diurnal rhythm of cortisol secretion which is what was detected in the current investigation [25].

In one particular study [26], the majority of a subject group consisting of older individuals with memory complaints presented an atypical cortisol profile that was characterised by a normal morning peak, with evening levels that did not reach the typical low nadir phase. This implies that atypical diurnal cortisol release may have significant effects on memory function.

Why is high evening cortisol in particular associated with recognition memory deficits? Recent studies have showed that stress induced in the morning, but not the afternoon, is associated with memory deficits [12,27,28]. In these studies, the observed impacts are due to acute manipulations of cortisol that are reversible. However, little is known about whether or not chronic stress is more detrimental at a certain time point during the day. Potentially, high cortisol levels at a time when they should be low may have negative impact on cognitive function. Alternatively, high evening cortisol may well interfere with sleep duration or quality, lack of which is associated with cognitive deficits [29]. In support of this, it was found that a lightening of sleep is accompanied by increases in cortisol, and the ability to enter REM (rapid eye movements) sleep cycles requires low cortisol levels at night, which are characteristic of this time of day [30].

The next question to answer is how does high cortisol_10 pm _selectively affect object recognition with no effect on other aspects of cognition? High cortisol_10 pm _may selectively induce transient changes in synaptic plasticity in specific neuronal circuitry such as perirhinal cortical synapses, which have been hypothesised as being an important brain region for visual object recognition [31]. These cortisol levels may be not high enough or long enough in duration to regulate synaptic plasticity in other neuronal circuitries, such as the hippocampus. There is evidence to support this hypothesis; in rodents and non-human primates, the perirhinal cortical region showed the highest density of glucocorticoid receptor (GR)-immunoreactive and GR-mRNA-containing cells than other brain regions [32,33]. Therefore, the pharmacological feature of GR activation in the perirhinal cortex may be a potential answer.

### Conclusion

Taken together, salivary cortisol level at the evening phase of the diurnal rhythm may have an important role in early deterioration of visual recognition memory in healthy female subjects. The loss of recognition memory, which is vital for everyday life, is a major symptom of the amnesic syndrome and early stages of Alzheimer's disease [31]. Therefore, this study will promote a potential physiologic marker of early deterioration of recognition memory. It will be of future interest to reveal whether this deterioration of recognition memory is due to neurological insults in specific brain regions.

## Methods

### Subjects

The subjects volunteered to participate in this study and had no medication history. Each subject filled in a questionnaire that provided information on health and lifestyle. This study was conducted in accordance with the Declaration of Helsinki.

### Cortisol Measurement

The subjects were asked to collect saliva samples at three particular time points (8 am, 2 pm and 10 pm) on two normal working days from two independent weeks. To avoid season-mediated light/dark cycle variance, saliva samples were collected between May – August of the year. Saliva was collected by means of a sterile microbiological swab (Bibby Sterilin Ltd, Stafford, UK). Subjects were asked to keep samples in a fridge until collection. Saliva was extracted from the swab by centrifugation at 2000 rpm at a temperature of 4°C for 6 minutes. Salivary cortisol was determined by means of a cortisol salivary immunoassay kit (Salimetrics Ltd, Pennsylvania, USA) and read in a luminometer. Each sample was determined in duplicate to identify potential sample loading errors.

### Cognitive Tasks

Before starting the experiment, the subjects were fully informed as to how to respond to the tasks by means of a visual instruction sheet. Subjects also completed a practice version of each task to ensure familiarisation with the procedure. The tasks were conducted with an Apple Macintosh computer using Macintosh stimulus presentation software (SuperLab; Cedrus, Wheaton, MD) [34] which presents images for the different cognitive tasks (for more details see Cho et al., 2000; Cho, 2001). All participants were tested in the afternoon between the hours of 2 pm and 5 pm to minimize possible differences in performance due to diurnal changes in cortisol.

Each visual task began with a 5 sec presentation of a black cross in the centre of the screen to hold the subject's attention. Test pictures were presented in a pseudorandom and counter-balanced order. The tasks required the subjects to choose the correct key responses. In the attention task, 'press key p' was required if the black triangle appeared at the top of the square, and 'press key q' if it appeared on the bottom. No response was required for any other image (i.e., neutral stimulation). The language task was based on one previously used [35] and involved an appropriate key response to presentation of a written word. Half of the words referred to man-made items (e.g., "scissors") while half were natural items (e.g., "cat"). Subjects were required to 'press key p' in response to a word referring to a natural item, and 'press key q' in response to a man-made item. No response was required for a neutral stimulation (black cross). All of the stimuli were highly familiar, concrete nouns according to the MRC psycholinguistic database [36].

Visual recognition memory was analysed using photographed images of novel and familiar objects. Images for the object recognition memory tasks were obtained from the websites  and . Familiar images were of everyday items (e.g., a chair or a shoe) while novel objects were obscure images the subjects were unlikely to have encountered ever before. The object discrimination task was based on the 'object familiarity detection task' used by Laatu and colleagues [37]. In this task the subject was presented with a sequence of familiar and novel objects that were shown in a pseudorandom and counterbalanced order. The subject was asked to decide whether the picture presented was a familiar object or not by pressing one of two keys on the computer keyboard. Therefore correct performance required decisions based on whether or not the presented shape has a representation in the subject's long-term memory [37]. For data analysis, responses to familiar images and responses to novel images were determined separately. The familiarity acquisition task used the same format as the object discrimination task but in this case, some images were repeated at later points in the sequence. Appearances of the same image were always parted by at least 7 other images (which didn't differ significantly between novel and familiar images). The reaction time to repeated presentations of novel images gave an indication whether or not the item was becoming familiar. A second difference between this task and the object discrimination task was that the subjects were asked to silently name the objects presented to them. If they could name them (familiar object) they were required to press one key, and if they were unable to name them (novel object) they were to press the other key. As this task concerned the reaction time to repeated novel images, this naming method was adopted in order to encourage the subjects to press the novel key if the image was indeed novel [37]. Essentially the subjects were still categorising the objects as either familiar or novel. By the fourth presentation of the novel image the subject begins to recognise the object and thus respond more quickly to the image. In this recognition task the subjects are unaware that they are encoding visual information about the objects. In addition, they are not aware that they are being tested for their memory of novel images. Therefore this task is an incidental encoding delay task.

In the spatial task, subjects were required to 'press key p' if the second presentation of a set of images were in the same position as when they first appeared, and 'press key q' if an image had changed its position. Repetitions of the same set of images were separated by a presentation of a black cross for a duration of 8 secs. The accuracy of correct responses (percentage) and reaction time (msec) were measured by the computer.

### Statistical analysis

Statistical analyses were carried out using Student's non-paired *t *test, the Mann-Whitney U-test and regression analysis. Data are presented as Mean ± s.e.m. *P *< 0.05 was taken as the level of significance throughout the analysis.

## Authors' contributions

HG carried out all of the experiments. DW confirmed statistics and coordinated the manuscript. KC conceived the study, supervised all experiments and coordinated the manuscript. All authors assisted with writing the manuscript.
